# Genetic Analysis of Virulence and β-Lactamase Determinants Related to β-Lactamase Inhibitors in *Pseudomonas aeruginosa* Strains from Nosocomial Infections

**DOI:** 10.3390/antibiotics15010016

**Published:** 2025-12-22

**Authors:** Gloria Luz Paniagua-Contreras, Elizabeth Olvera-Navarro, Jennefer Paloma Herrera-Gabriel, Laura Verónica González-Vega, Luis Rey García-Cortés, Moisés Moreno-Noguez, Héctor Martínez-Gregorio, Felipe Vaca-Paniagua, Ana María Fernández-Presas, Eric Monroy-Pérez

**Affiliations:** 1 Facultad de Estudios Superiores Iztacala, Universidad Nacional Autónoma de México, Tlalnepantla de Baz 54090, Mexico; 2 Coordinación de Investigación del Estado de México Oriente, Insitituto Mexicano del Seguro Social, Tlalnepantla de Baz 50090, Mexico; 3 Unidad de Biomedicina, Facultad de Estudios Superiores Iztacala, Universidad Nacional Autónoma de México, Tlalnepantla de Baz 54090, Mexico; 4 Laboratorio Nacional en Salud, Diagnóstico Molecular y Efecto Ambiental en Enfermedades Crónico-Degenerativas, Facultad de Estudios Superiores Iztacala, Universidad Nacional Autónoma de México, Tlalnepantla de Baz 54090, Mexico; 5 Departamento de Microbiología y Parasitología, Facultad de Medicina, Universidad Nacional Autónoma de México, Ciudad de México 04510, Mexico

**Keywords:** virulence genes, β-lactamase genotypes, β-lactamase inhibitors

## Abstract

**Background/Objectives**: The emergence of hypervirulent *Pseudomonas aeruginosa* strains resistant to β-lactamase inhibitor antibiotics is a critical health problem as they impede the treatment of infections. The objective of this study was to determine the different molecular arrangements of the virulence genotype related to β-lactamase genotype and the resistance phenotype to a combination of β-lactam antibiotics and β-lactamase inhibitors, and the phylogroups in *P. aeruginosa* strains isolated from patients with healthcare-associated infections and community-acquired infections. **Methods**: *P. aeruginosa,* virulence genes, β-lactamase genes and phylogroups were identified using polymerase chain reaction. Resistance to β-lactam antibiotics and β-lactamase inhibitors was determined using the disk diffusion method. The MIC determination of ticarcillin/clavulanic acid and piperacillin/tazobactam was performed using the MIC test strip for antimicrobial susceptibility testing. **Results**: In total, 124 *P. aeruginosa* strains from patients with healthcare-associated (67/124) and community-acquired infections (57/124) were analyzed. Most strains from patients with healthcare-associated infections and community-acquired infections harbored genes for proteases (*aprA*), phospholipases (*pIcH* and *pIcN*), elastases (*lasA* and *lasB*), rhamnolipids (*rhLA*), quorum-sensing system (*lasI* and *rhII*), and β-lactamase (*bla_oxa-4_*, *bla_oxa-1_*, and *bla_GES_*). In total, 100% (124/124) and 99.1% (123/124) of the strains isolated from patients with healthcare-associated and community-acquired infections were resistant to the β-lactamase inhibitor antibiotics, amoxicillin/clavulanic acid and ampicillin/sulbactam, respectively, while 54% (67/124) of the strains were resistant to piperacillin/tazobactam. Phylogroup 1 (22/124) was detected more frequently among the strains in relation to phylogroup 2 (8/12). **Conclusions**: We demonstrated different association profiles of virulence genotype related to the β-lactamase genotype, the β-lactamase inhibitor resistome, phylogroups, and clinical origin of the strains. Therefore, medical treatment regimens against infections caused by *P. aeruginosa* should be improved.

## 1. Introduction

*Pseudomonas aeruginosa* is a gram-negative nosocomial pathogen that exhibits high multidrug resistance, which complicates medical treatment and increases the morbidity and mortality rates of patients with healthcare-associated infections [1,2] such as meningitis, pneumonia, bacteremia, respiratory infections, urinary tract infections (UTIs), catheter-associated infections, and surgical wound infections [3,4,5,6,7]. Owing to the increase in the prevalence of multidrug-resistant (MDR) and extensively drug-resistant hospital-acquired strains of *P. aeruginosa* [8,9], the World Health Organization has included it in the list of critical pathogens for which development of new antibiotics is urgently required [10]. The International Network for Optimal Resistance Monitoring reported that the rate of occurrence of MDR *P. aeruginosa* associated with different types of infections during 2025–2016 in different hospital centers in the USA ranged from 11.5 to 20.7% [11], whereas the Centers for Disease Control of the USA reported an incidence of 32,600 cases and 2500 deaths caused by MDR *P. aeruginosa* strains in hospitalized patients in 2019 [12]. The pathogenicity of *P. aeruginosa* during infections is due to the presence of different virulence factors that encode the alginate capsule (*algD*), adhesins (*pilA*), biofilms (*ndvB*), elastases (*lasA* and *lasB*), alkaline protease (*aprA*), phospholipases (*pIcH* and *pIcN*), rhamnolipids (*rhLA*), and exotoxins of the type 3 secretion system (T3SS; *exoT, exoY, exoS* and *exoU*) that destroy host tissues and favor the invasion and acuity of infections [13,14]. Three phylogroups have been identified in *P. aeruginosa* strains, those of clade 1 produce the T3SS ExoS effector, while most of those belonging to clade 2 produce the T3SS ExoU effector [15]. The expression of most virulence factors, including biofilm formation, is regulated by the quorum-sensing systems (*lasI* and *rhII*) [16].

The emergence of MDR *P. aeruginosa* strains harboring genes for resistance to aminoglycosides, tetracyclines, fluoroquinolones, carbapenemases, and extended-spectrum β-lactamases (BLES) [17,18,19] is a serious health problem that significantly increases healthcare costs, hospitalization times, and patient mortality [20].

Considering the significant increase in the number of hospital-acquired infections caused by MDR *P. aeruginosa* strains, which are producers of BLES [21,22], the use of combinations of β-lactam antibiotics with β-lact amase inhibitors has become necessary to treat patient infections [23,24,25]. However, the emergence of strains resistant to these antimicrobial agents has become increasingly common [26].

In Mexico, multidrug-resistant (MDR) and hypervirulent *P. aeruginosa* is the second most prevalent opportunistic pathogen associated with nosocomial infections [27,28], therefore, the implementation of new antibiotics and new combinations of antibiotics with β-lactamase inhibitors in public hospitals is essential to reduce mortality rates. In this regard, the purpose of this study was to investigate the different molecular arrangements of the virulence genotype related to β-lactamase genotype and the resistance phenotype to a combination of β-lactam antibiotics and β-lactamase inhibitors and the phylogroups in *P. aeruginosa* strains isolated from patients with healthcare-associated infections and community-acquired infections.

## 2. Results

### 2.1. Distribution of Virulence Genes with Respect to Strain Origin

Fifty-four percent (67/124) of the strains were isolated from hospitalized patients with healthcare-associated infections (pneumonia, bacteremia, and wound infections) and 46% (57/124) from those with community-acquired infections (respiratory tract infections, catheter-associated infections, and UTIs; Table 1). Overall, the distribution of virulence genes in the strains was independent of the type of infection (*p* < 0.05; Table 1). All strains isolated from patients with healthcare-associated and community-acquired infections car-ried rhamnolipid (*rhlA*) and quorum-sensing (*lasI* and *rhll*) genes, while all strains from patients with wound (27/27), respiratory (10/10), and catheter-associated infections (10/10) harbored alkaline protease (*aprA*)- and phospholipase (*pIcH*)-coding genes. The *pIcN* (phospholipase) gene was identified in most strains from patients with wound infection (25/27), bacteremia (22/24), respiratory infection (9/10), catheter-associated infection (9/10), UTI (33/37), and pneumonia (14/16). In contrast, all strains isolated from patients with pneumonia (16/16), bacteremia (24/24), respiratory infection (10/10), and catheter-associated infection (10/10) harbored the elastase (*lasA*)-coding gene, whereas the other elastase gene (*lasB*) was detected more frequently in strains isolated from patients with community-acquired infections, including respiratory infection (9/10), catheter-associated infection (9/10), and UTI (31/37).

### 2.2. Frequency of β-Lactamase Genotype and Resistance to β-Lactamase Inhibitor Antibiotics in the Strains

All strains isolated from patients with healthcare-associated infections, such as pneumonia (16/16) and wound infections (27/27), and from strains isolated from community-acquired infections, such as respiratory infections (10/10), harbored *bla_oxa-_*_2_ (Table 2). High percentages of *bla_oxa-_*_4_ and *bla_GES_* were also found in most strains from patients with healthcare-associated infections (pneumonia, bacteremia, and wound infections) and in strains from patients with community-acquired infections (respiratory infections, catheter-associated infections, and UTI). *bla_oxa-_*_1_ was most frequently detected in strains from patients with UTI (14/37), wound infections (8/27), and respiratory infections (3/10), whereas *bla_SHV_* was most frequently identified in strains isolated from patients with catheter-associated infections (3/10), UTI (7/37), pneumonia (3/16), and wound infections (5/27).

All strains isolated from patients with healthcare-associated infections, such as pneumonia (16/16) and wound infection (27/27), and from patients with community-acquired infections, such as respiratory infection (10/10), catheter-associated infection (10/10), and UTI (37/37), were resistant to β-lactamase inhibitor antibiotics (amoxicillin/clavulanic acid and ampicillin/sulbactam; Table 2). Similarly, 100% (24/24) and 95.8% (23/24) of the bacteremia strains were resistant to ampicillin/sulbactam and amoxicillin/clavulanic acid, respectively. In contrast, most strains isolated from patients with respiratory tract infections (8/10), catheter-associated infections (7/10), and wound infections (18/27) were resistant to piperacillin/tazobactam (Table 2). A significant association was observed between the detection frequencies of *bla_CITM_* and *bla_GES_*, as well as between the piperacillin/tazobactam resistance phenotype of the strains and the type of infection in the patients (Table 2).

### 2.3. Frequency of Virulence Genes According to β-Lactamase and β-Lactamase Inhibitor Genotypes

Overall, a significant correlation was observed between the frequency of the virulence genotype and the β-lactamase genotype and resistance to β-lactamase inhibitors in strains isolated from patients with healthcare-associated infections (67/124) (*p* = 0.0004, Table 3). High correlation percentages with the resistance phenotype to β-lactamase inhibitor antibiotics (amoxicillin/clavulanic acid and ampicillin/sulbactam) and with β-lactamase genes (*bla_oxa-_*_2_, *bla_oxa-_*_4_ and *bla_GES_*; Table 3), ranging from 55.2% (37/67) to 100% (67/67) were found for genes encoding protease (*aprA*), phospholipase (*plcH* and *plcN*), elastase (*lasA* and *lasB*), rhamnolipid (*rhLA*), and the quorum-sensing system (*lasl* and *rhll*). Similarly, a correlation was found between the virulence genotype frequency (proteases, phospholipases, elastases, rhamnolipids, and quorum sensing) of strains and resistance to the β-lactamase inhibitors, piperacillin/tazobactam, ranging from 38.8% (26/67) to 50.7% (34/67).

Regarding strains isolated from patients with community-acquired infections (57/124), a significant correlation was found between the frequency of the virulence genotype and the β-lactamase genotype and resistance to β-lactamase inhibitors (*p* = 0.0004, Table 4). In general, the virulence genotype (*aprA*, *plcH* and *plcN*, *lasA* and *lasB*, *rhLA*, *lasI*, and *rhII*) showed high correlation with β-lactamase inhibitor antibiotics (amoxicillin/clavulanic acid and ampicillin/sulbactam) and with the β-lactamase genotype (*bla_oxa-_*_2_, *bla_oxa-_*_4_, and *bla_GES_*; Table 4), with percentages ranging from 52.6% (30/57) to 100% (57/57).

### 2.4. Distribution of Phylogroups and MIC for β-Lactamase Inhibitor Antibiotics According to the Origin of the Strains

Overall, phylogroup 1 was the most frequent (22/124) among the strains (Table 5), particularly in strains from patients with UTI (8/37), wound infection (4/27), and bacteremia (4/24), while phylogroup 2 (8/124) was more frequent in strains from patients with catheter-associated infection (2/10), wound infection (2/27), and bacteremia (2/24). The phylogroup was not identified in 75.8% (94/124) of the strains.

Most strains (123/124) from healthcare-associated infections and community-acquired infections were resistant to the ticarcillin/clavulanic acid combination with an MIC > 256 µg/mL (Table 5), while 54% (67/124) were resistant to the piperacillin/tazobactam combination with an MIC > 256 µg/mL and 46% (57/124) were susceptible with MICs in the range of 3–96 µg/mL (Table 5).

### 2.5. Analysis of the overall distribution of virulence and resistance to β-lactams

**Figure 1:**
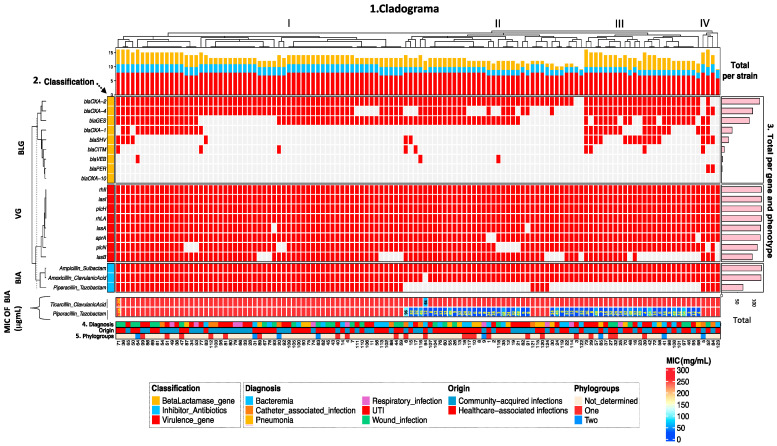
Unsupervised hierarchical clustering of *P. aeruginosa* strains according to virulence genotype, β-lactamase gene, β-lactamase inhibitor antibiotic resistance phenotype, and clinical features. The heat map is divided into panels. Top panel: (1) general cladogram of genotype and phenotype distribution among strains belonging to groups I, II, III, and IV. Left panel: (2) β-lactamase genotype (BLG), virulence genotype (VG), β-lactamase inhibitor antibiotic resistance phenotype, and MIC of β-lactamase inhibitor antibiotic among strains (BIA). Right panel: (3) absolute frequency for each gene and the resistance phenotype; bottom panel: (4) clinical diagnosis and sample origin and (5) phylogroups assigned to the strains. The presence of a gene is shown in red and its absence in gray.

Unsupervised hierarchical clustering analysis showed wide distribution and diversity of genotype and phenotype frequencies among *P*. aeruginosa strains. The general cladogram was divided into four main groups based on virulence genes, β-lactamase genes, resistance phenotype to β-lactamase inhibitor antibiotics, phylogroups, and the clinical origin of the strains (Figure 1). Group I was the largest and was composed of 59 strains (range of strains 71–59), group II was composed of 37 strains (range of strains 6–122), group III comprised 24 strains (range of strains 73–68), and group IV comprised four strains (range of strains 5–123). The β-lactamase genes showed high variability in strains of the four groups, particularly *bla_OXA-2_*, *bla_OXA-4_*, and *bla_GES_*, which were detected most frequently. In contrast, virulence genes of the quorum sensing system (*lasI* and *rhlI*), rhamnolipids (*rhlA*), elastases (*lasA* and *lasB*), phospholipases (*plcH* and *plcN*), and alkaline proteases (*aprA*) were detected in almost all strains belonging to the four groups, highlighting their conserved role in virulence regulation. Overall, almost all strains in all four groups presented a β-lactamase inhibitor antibiotic (amoxicillin/clavulanic acid and ampicillin/sulbactam) resistance phenotype profile, whereas the resistance profile to piperacillin/tazobactam was more frequent in strains belonging to group I. In group I, subgroups of strains (No. 109, 105, 100, 90, 75, 74, 63, 62, 45, 43, 40, 14, 4, and 7) and (No. 112, 103, 96, 81, 80, 79, 66, 33, 28, and 31) were identified with identical virulence genotype characteristics, β-lactamase genotype, and resistance phenotype to β-lactamase inhibitors of antibiotics, but of different origin, diagnosis, and phylogroup. The same occurred with other groups of strains in groups I, II, and III. When molecular data was integrated with clinical parameters (diagnosis and origin of infection), clustering did not reveal any clear associations, suggesting that genotype-phenotype diversity is largely independent of clinical presentation.

### 2.6. Patterns of Association of Virulence Genotype and β-Lactamases with the Phenotype of Resistance to β-Lactamase Inhibitor Antibiotics

Fifty-five different association patterns of virulence genes related to β-lactamase genes and β-lactamase-inhibiting antibiotics were identified among the strains (Table 6). Pattern No. 1 (*aprA*/*pIcH*/*pIcN*/*lasA*/*lasB*/*rhLA*/*lasI*/*rhII*/*bla_OXA-_*_2_/*bla_OXA-_*_4_/*bla_GES_*/Am-Sul/Amox-Ac.Clavul/Piper-Tazo) was the most abundant, comprising 14 strains (11.3%), followed by pattern No. 2 (*aprA*/*pIcH*/*pIcN*/*lasA*/*lasB*/*rhLA*/*lasI*/*rhII*/*bla_OXA-_*_2_/*bla_OXA-_*_4_/Am-Sul/Amox-Ac.Clavul/Piper-Tazo) with 10 strains (8%).

## 3. Discussion

Hospital-acquired infections caused by MDR *P. aeruginosa* represent a serious global health problem, which has increased healthcare cost and mortality rates [29]. In the USA, the average mortality rate of patients infected with MDR *P. aeruginosa* (n = 524) from 78 hospitals was 23.7%, with an average hospital stay of 39.7 days and average cost per case of USD 124,335 [30]. In this study, we analyzed a group of *P. aeruginosa* strains isolated from patients hospitalized with healthcare-associated infections (67/124), primarily bacteremia (27/67) and wound infections (27/67), as well as another group of strains isolated from outpatients with community-acquired infections (57/124), which predominantly included UTIs (37/67). Pneumonia and bacteremia are two of the most prevalent healthcare-associated infections caused by *P. aeruginosa*, resulting in high mortality rates in hospitals [31,32]. Among community-acquired infections, catheter-associated UTIs are the most common conditions observed in outpatients [33].

The acuteness of hospital and community infections caused by *P. aeruginosa* occurs because of the large number of virulence factors that favor adhesion, colonization, tissue degradation, and evasion of the host immune response [34]. In this study, the distribution frequencies of the virulence genotype among strains isolated from patients with healthcare-associated infections (67/124) and community-acquired infections (57/124) were similar, with percentages of protease (*aprA*), phospholipase (*pIcN* and *pIcN*), elastases (*lasA*), rhamnolipids (*rhIA*), and quorum-sensing (*lasI* and *rhII*) genes ranging from 87.5% to 100% in strains from hospitalized patients (having pneumonia, bacteremia, and wound infection) and outpatients (having respiratory infection or catheter-associated infection and UTI). In general, the frequency of virulence genes detected in hospital and community strains of *P. aeruginosa* (*aprA*, *pIcN*, *pIcN*, *lasA*, *lasB*, *rhIA*, *lasI*, and *rhII*) is similar to that mentioned in other studies in strains isolated from different infectious processes [35,36,37,38,39]. The high frequency of these virulence markers in strains from patients with healthcare-associated infections and community-acquired infections can explain the increase in mortality rates; this is because alkaline protease (AprA) and elastase B (LasB) are involved in cystic fibrosis in patients with respiratory infections [40], phospholipase (PicH) promotes tissue damage and inflammation [41], rhamnolipids (RhIA) participate in biofilm formation, which contribute to the evasion of host’s immune response and protection against antibiotics [42], and quorum-sensing systems (LasI and RhII) regulate the expression of numerous virulence factors that favor colonization, invasion, and tissue damage, causing acute or chronic infections [43].

The emergence of hospital-acquired MDR strains of *P. aeruginosa* is a major factor reducing medical treatment options and increasing mortality rates [44]. In a previous study, we analyzed the molecular properties of virulence related to the antibiotic resistance phenotype in the same *P. aeruginosa* strains (n = 124) and found that 100% (n = 124) of the strains were MDR with high percentages of resistance to β-lactam antibiotics (ampicillin, carbenicillin, cephalotin, and cefotaxime) [45]. Therefore, in continuation of our research, we analyzed the resistance phenotype to β-lactamase inhibitor antibiotics. Overall, our results showed a wide distribution of the BLES genotype among the strains; the highest percentages were observed for the *bla_OXA-2_, bla_OXA-4_*_,_ and *bla_GES_* genes in most strains from patients with healthcare-associated infections (pneumonia, bacteremia, and wound infection) and acquired community infections (respiratory infection, catheter-associated infection, and UTI), while lower percentages were found for *bla_OXA-1_* and *bla_SHV_*, mainly for strains isolated from patients with UTI, catheter-associated infection, wound infection, and pneumonia. The percentages of *bla_OXA-2_, bla_OXA-4_,* and *bla_GES_* genes detected in strains isolated from patients with healthcare-associated and community-acquired infections are higher than those described in 31,061 *P. aeruginosa* strains isolated from patients in 99 countries using the NCBI Microbial Browser for Identification of Genetic and Genomic Elements (MicroBIGG-E) database [46]. The percentages of *bla_SHV_* and *bla_OXA-_*_2_ in our strains were also higher than those described in a large study conducted in 14 Arab countries between 2011 and 2018 [47]. Different combinations of antibiotics with β-lactamase inhibitors have been used to counteract infections by BLES-producing *P. aeruginosa* strains; however, strains resistant to these combinations have been detected over time [48]. The results from the resistogram analysis showed that all strains (124/124) from healthcare-associated infections (pneumonia, bacteremia, and wound infection) and community-acquired infections (respiratory infection, catheter-associated infection, and UTI) were resistant to the ampicillin/sulbactam combination. The same was observed with amoxicillin/clavulanic acid, and with the combination of ticarcillin/clavulanic acid, where almost all hospital- and community-acquired strains (123/124) were resistant to this combination (MIC > 256 µg/mL), except for one strain isolated from a patient susceptible to bacteremia (MIC = 64 µg/mL). In contrast, 54% (67/124) of the strains were resistant to piperacillin/tazobactam (CMI > 256 μg/mL), with higher percentages of resistance observed in strains isolated from patients with respiratory (8/10), catheter-associated (7/10), and wound infections (18/27). The low effectiveness of amoxicillin/clavulanic acid, ticarcillin/clavulanic acid and ampicillin/sulbactam and the moderate effectiveness of piperacillin/tazobactam against hospital and community strains of *P. aeruginosa* may be due to the high frequency of the genes, *bla_oxa-2_* (119/124), *bla_oxa-4_* (97/124), *bla_oxa-1_* (33/124), and *bla_GES_*, in which case, clavulanic acid, sulbactam, and tazobactam are mainly effective against class A β-lactamases (TEM-1, TEM-2, and SHV-1) [49]. Around 120 different β-lactamases have been identified in *P. aeruginosa* [50], which could explain the high resistance observed in this study to combinations of β-lactamase inhibitor antibiotics; hence, new combinations such as ceftazidime/avibactam, meropenem/vaborbactam, and imipenem/relebactam may be used as alternatives against acute hospital infections caused by *P. aeruginosa* [51,52]. The resistance rates for amoxicillin/clavulanic acid, ampicillin/sulbactam, and piperacillin/tazobactam found in this study in strains isolated from patients with healthcare-associated infections and community-acquired infections are higher than those described in other countries for *P. aeruginosa* strains isolated from hospitalized patients [53,54,55]. The percentages of resistance to piperacillin/tazobactam found in this study are also similar to those described in *P. aeruginosa* strains isolated in Mexico and other Latin American countries [56]. Due to the significant increase in resistance to β-lactamase inhibitor antibiotics in hospital strains of *P. aeruginosa* in Mexico, it is essential to implement next-generation β-lactamase inhibitor antibiotics in the Mexican public health sector.

It is noteworthy that overall a high correlation was found between protease (*aprA*), phospholipase *(pIcH* and *pIcN*), elastase (*lasA* and *lasB*), rhamnolipid (*rhLA*) and quorum-sensing system (*lasI* and *rhII*) genes and the resistance phenotype to the β-lactamase inhibitor antibiotics, amoxicillin/clavulanic acid and ampicillin/sulbactam; high correlation was also found with the β-lactamase genotype, *bla_oxa-4_, bla_oxa-1,_* and *bla_GES_*_,_ in strains isolated from patients with healthcare-associated infections (67/124) and community-acquired infections (57/124). The high frequency of virulome correlation with the β-lactamase genotype and β-lactamase inhibitor resistance phenotype detected in hospital and community strains is an important factor that can dramatically increase patient mortality rates. The emergence of *P. aeruginosa* strains with difficult-to-treat resistance in Mexico and other countries, which are resistant to all first-line antibiotics (carbapenems, combinations of β-lactams and β-lactamase inhibitors, and fluoroquinolones) [56,57,58,59] and also present high molecular characteristics of hypervirulence [60,61], currently represents a serious challenge for health systems worldwide. Hence, new treatment options that may help reduce mortality rates are required.

In this study, phylogroup 1 was identified more frequently (22/124) among strains isolated from patients with healthcare-associated infections (11/22) and community-acquired infections (11/22), while phylogroup 2 was less frequent (8/124). In Mexico, phylogroup 2 has been described as more abundant than phylogroups 1 and 3 in hospital strains isolated from burn patients over a 10-year period [62].

Unsupervised hierarchical clustering analysis showed a wide distribution and diversity of virulence genotype and β-lactamase genes, related to the resistome, phylogroup, and origin of strains isolated from patients with healthcare-associated and community-acquired infections. The strains were distributed into four main groups (I, II, III, and IV) and presented different genotype-phenotype association profiles, phylogroup, and clinical origin, with group I presenting the highest frequency of strains with the same virulence and antimicrobial resistance properties; in other words, strains no. 109 (UTI), 105 (wound infection), 100 (bacteremia), 90 (pneumonia), 75 (pneumonia), 74 (respiratory infection), 63 (wound infection), 62 (bacteremia), 45 (wound infection), 43 (wound infection), 40 (UTI), 14 (bacteremia), 4 (respiratory infection), and 7 (wound infection) presented the same virulence and β-lactamases genotypes related to the resistogram (amoxicillin/clavulanic acid, ampicillin/sulbactam, and piperacillin/tazobactam, and with the MIC of ticarcillin/clavulanic acid and piperacillin/tazobactam); the same was observed with other groups of strains belonging to groups II and III, where phylogroup 1 was more frequent than phylogroup 2.

Different patterns of association were identified between virulence and β-lactamase genes with the phenotype of resistance to β-lactamase inhibitor antibiotics, and with phylogroups in strains from infected patients, because of which we speculated the presence of different virulome expression profiles in strains associated with hospital and community infections; hence, in future research it will be interesting to analyze the expression of virulence genes in the strains using in vitro models of infection in human epithelial cell lines, pulsed field gel electrophoresis, serotyping, and massive genome sequencing of these strains should be performed to provide more information on the molecular composition of the virulome and the antibiotic resistance genotype in strains isolated from patients with healthcare-associated and community-acquired infections.

## 4. Methods

### 4.1. Origin of the Strains

Sixty-seven *P. aeruginosa* strains isolated from patients with ongoing healthcare-associated infections (bacteremia, pneumonia, and wound infections) were analyzed after they were admitted to the hospital for the treatment of other comorbidities, such as chronic kidney failure, diabetes mellitus, obstructive pulmonary disease, and high blood pressure. Fifty-seven strains from non-hospitalized patients with community-acquired infections (respiratory, catheter-associated, and UTIs) were also studied. The strains were collected from the Microbiology Laboratory of Regional General Hospital No. 72 of the Mexican Social Security Institute, located in the municipality of Tlalnepantla de Baz, State of Mexico, Mexico, between September 2022 and December 2023. Written informed consent was obtained from all study participants. This study was approved by the Institutional Ethics Committee (R-2024-1406-014). *Pseudomonas aeruginosa* strains were identified using an automated VITEK 2 Compact (bioMérieux, Marcy l’Etoile, France) and confirmed using polymerase chain reaction (PCR).

### 4.2. Determination of Resistance to β-Lactam Antibiotics with β-Lactamase Inhibitors

Resistance of *P. aeruginosa* strains to the antibiotics, amoxicillin + clavulanic acid (20 + 10 micrograms), ampicillin + sulbactam (10 + 10 micrograms), and piperacillin + tazobactam (100 + 10 micrograms) was determined using the disk diffusion method (Thermo Scientific™ Oxoid™, Scheepsbouwersweg 1B, 1121 PC, Landsmeer, The Netherlands). *E. coli* ATCC 25922 and *P. aeruginosa* ATCC 15692 were used as controls for reproducibility. Results were interpreted using the criteria established by the Clinical and Laboratory Standards Institute [63].

### 4.3. Determination of the MIC of β-Lactamase Inhibitor Antibiotics

The MIC determination of ticarcillin/clavulanic acid (0.016–256 μg/mL) and piperacillin/tazobactam (0.016–256 μg/mL) was performed according to the MTS™ quantitative assays using the MIC test strip for antimicrobial susceptibility testing (Liofilchem^®^ Inc., Waltham, MA, USA), following the manufacturer’s instructions.

### 4.4. DNA Extraction

DNA was extracted from the strains using the boiling method [64]. Strains were plated using the cross-streak method on cetrimide agar (MCD LAB, Tlalnepantla de Baz, Mexico) and incubated at 37 °C for 24 h. Six colonies approximately 2 mm in diameter were removed from the pure cultures of each strain using a sterile loop and placed in Eppendorf tubes containing 1.5 mL of sterile deionized water. The tubes were boiled for 20 min and incubated on ice for 10 min. Finally, they were centrifuged at 10,000 rpm for 10 min and the supernatant containing the DNA was separated and stored in another Eppendorf tube at −20 °C.

### 4.5. Strain Identification

*P. aeruginosa* was identified by amplifying its *16S rDNA* gene using PCR [65]. The final volume of the PCR mixture was 25 μL, which consisted of 12.5 μL of Taq DNA polymerase master mix RED (Ampliqon, Copenhagen, Denmark), 3 μL of template DNA (100 ng), 1 μL of forward primer, 1 μL of reverse primer (10 pmol, Integrated DNA Technologies, San Diego, CA, USA), and 7.5 μL of nuclease-free water. The amplification conditions were as follows: initial denaturation at 95 °C for 2 min, followed by 25 cycles at 94 °C for 25 s, 58 °C for 40 s, and 72 °C for 40 s. Finally, an extension was performed at 72 °C for 1 min. *P. aeruginosa* strain ATCC 27853 was used as the positive control.

### 4.6. Identification of Virulence Genes

Single-plex PCR to identify the genes encoding alkaline protease (*aprA*), phospholipase (*pIcH* and *pIcN*), elastase (*lasA* and *lasB*), rhamnolipid (*rhLA*), and the quorum-sensing system (*lasI* and *rhII*) were performed as described previously [35,36,37,66]. The final volume per reaction mix for each uniplex PCR assay was 25 μL, which consisted of 12.5 μL of Taq DNA polymerase master mix RED (Ampliqon, Copenhagen, Denmark), 3 μL of template DNA (100 ng), 1 μL of forward primer, 1 μL of reverse primer (10 pmol, Integrated DNA Technologies), and 7.5 μL of nuclease-free water. *P. aeruginosa strain* ATCC 27853 was used as the positive control.

### 4.7. Detection of BLES Genes

The PCR conditions and oligonucleotides used to identify *bla_SHV_* and *bla_TEM_* [67], *bla_OXA-1_*, *bla_OXA-2_*, *bla_OXA-4_*, *bla_OXA-10_*, *bla_GES_*, *bla_PER_*, and *bla_VEB_* [68] have been described previously. For each uniplex PCR assay, a final reaction volume of 25 μL was used, which consisted of 12.5 μL of Taq DNA polymerase master mix RED (Ampliqon, Copenhagen, Denmark), 3 μL of template DNA (100 ng), 1 μL of forward primer, 1 μL of reverse primer (10 pmol, Integrated DNA Technologies), and 7.5 μL of nuclease-free water. The PCR amplicons were stained with Midori Green (Nippon Genetics, Düren, Germany) after electrophoresis on 2% agarose gels, which were photographed under UV using GEL LOGIC 100 (Kodak, Carestream Molecular Imaging, Rochester, NY, USA).

### 4.8. Identification of Phylogroups

The phylogroups of *P. aeruginosa* were identified by conventional PCR amplifying the *exoS* and *PA14300* genes as previously described [69]. The final volume per reaction mix for each uniplex PCR assay was 25 μL, which consisted of 12.5 μL of Taq DNA polymerase master mix RED (Ampliqon, Copenhagen, Denmark), 3 μL of template DNA (100 ng), 1 μL of forward primer, 1 μL of reverse primer (10 pmol, Integrated DNA Technologies), and 7.5 μL of nuclease-free water. The amplification conditions included an initial denaturation at 95 °C for 3 min, followed by 30 cycles at 95 °C for 30 s, 59 °C for 30 s and 72 °C for 75 s, with a final extension at 72 °C for 5 min.

### 4.9. Statistical Analysis

The chi-square test was performed using SPSS (version 20.0; SPSS Inc., Chicago, IL, USA) to establish the association between the frequency of virulence genotype, the presence of β-lactamase genes, and the phenotype of resistance to β-lactamase inhibitor antibiotics in strains isolated from patients with healthcare-associated and community-acquired infections, considering their respective diagnoses (*p* < 0.05).

### 4.10. Unsupervised Hierarchical Clustering

*P. aeruginosa* strains were systematically clustered according to the frequency of virulence genes, β-lactamase genes, resistance phenotype to β-lactamase inhibitor antibiotics, phylogroups, and infection type using unsupervised hierarchical clustering with Gower’s similarity coefficient [70]. The analysis was based on a categorical data matrix constructed in R (v3.6.1) with the cluster package (v2.1.0), which integrated the presence or absence of virulence and β-lactamase genes, the strains’ resistance phenotype to β-lactamase inhibitor antibiotics, phylogroups, and the diagnosis and origin of patients with healthcare-associated infections and community-acquired infections. The distance between strains was calculated based on the overall similarity coefficient, which was used to estimate the maximum possible absolute discrepancy between each pair of matched strains. After calculating the distances, mutually exclusive groups were clustered using Ward’s method in R [71]. Strains were visualized using a genotype-phenotype distribution plot and infection type, and a dendrogram was constructed using a complex heat map (v2.24.1).

## 5. Conclusions

The results of this study provide new information regarding the extensive composition and distribution of the virulome related to β-lactamase genotype and resistome among *P. aeruginosa* strains isolated from patients with healthcare-associated and community-acquired infections. The information obtained regarding the different profiles consisting of virulence genes, β-lactamase genes, and β-lactamase inhibitors in the strains may be used to improve therapeutic options to help reduce patient mortality caused by *P. aeruginosa* infections.

## Figures and Tables

**Table 1:** Frequency of virulence genes in the strains.

Strain Origin (n = 124)	Infection	Function (Gene)							
		Protease	Phospholipases		Elastases		Rhamnolipids	Quorum-Sensing	
		*aprA* No. (%)	*pIcH* No. (%)	*plcN* No. (%)	*lasA* No. (%)	*lasB* No. (%)	*rhlA* No. (%)	*lasI* No. (%)	*rhII* No. (%)
Healthcare associated infections (n = 67)	Pneumonia (n = 16)	15 (93.7)	16 (100)	14 (87.5)	16 (100)	12 (75)	16 (100)	16 (100)	16 (100)
	Bacteremia (n = 24)	23 (95.8)	24 (100)	22 (91.6)	24 (100)	14 (58.3)	24 (100)	24 (100)	24 (100)
	Wound infection (n = 27)	27 (100)	27 (100)	25 (92.5)	26 (96.2)	21 (77.7)	27 (100)	27 (100)	27 (100)
Community-acquired infections (n = 57)	Respiratory infection (n = 10)	10 (100)	10 (100)	9 (90)	10 (100)	9 (90)	10 (100)	10 (100)	10 (100)
	Catheter associated infection (n = 10)	10 (100)	10 (100)	9 (90)	10 (100)	9 (90)	10 (100)	10 (100)	10 (100)
	UTI (n = 37)	36 (97.3)	37 (100)	33 (89.1)	36 (97.3)	31 (83.7)	37 (100)	37 (100)	37 (100)
	*p*-value	0.2903	1	0.995	1	0.5	1	1	1
	Total, No. (%)	121 (97.5)	124 (100)	112 (90.3)	122 (98.3)	96 (77.4)	124 (100)	124 (100)	124 (100)

**Table 2:** Frequency of the phenotype of resistance to β-lactamase inhibitor antibiotics related to β-lactamase genes in the strains.

Strain Origin (n = 124)	Infection	Phenotype of Resistance to β-Lactamase Inhibitor Antibiotics			β-Lactamase Genes								
		Amoxicillin/ Clavulanic Acid	Ampicillin/ Sulbactam	Piperacillin /Tazobactam	*bla_SHV_* No. (%)	*bla_CITM_* No. (%)	*bla_OXA-_*_1_ No. (%)	*bla_OXA-_*_2_ No. (%)	*bla_OXA-_*_4_ No. (%)	*bla_OXA-_*_10_ No. (%)	*bla_GES_* No. (%)	*bla_PER_* No. (%)	*bla_VEB_* No. (%)
Healthcare associated infections (n = 67)	Pneumonia (n = 16)	16 (100)	16 (100)	6 (37.5)	3 (18.7)	0 (0)	4 (25)	16 (100)	12 (75)	0 (0)	10 (62.5)	0 (0)	0 (0)
	Bacteremia (n = 24)	23 (95.8)	24 (100)	10 (41.6)	4 (16.6)	0 (0)	2 (8.3)	22 (91.6)	17 (70.8)	0 (0)	15 (62.5)	0 (0)	0 (0)
	Wound infection (n = 27)	27 (100)	27 (100)	18 (66.6)	5 (18.5)	1 (3.7)	8 (29.6)	27 (100)	25 (92.5)	0 (0)	25 (92.5)	1 (3.7)	2 (7.4)
Community-acquired infections (n = 57)	Respiratory infection (n = 10)	10 (100)	10 (100)	8 (80)	0 (0)	0 (0)	3 (30)	10 (100)	7 (70)	0 (0)	5 (50)	0 (0)	0 (0)
	Catheter associated infection (n = 10)	10 (100)	10 (100)	7 (70)	3 (30)	3 (30)	2 (20)	8 (80)	7 (70)	0 (0)	7 (70)	1 (10)	1 (10)
	UTI (n = 37)	37 (100)	37 (100)	18 (48.6)	7 (18.9)	5 (13.5)	14 (37.8)	36 (97.3)	29 (78.3)	0 (0)	25 (67.5)	0 (0)	0 (0)
	*p*-value	0.438	1	**0.001**	0.667	**0.024**	0.191	0.096	0.317	0 (0)	**0.047**	0.216	0.141
	Total No. (%)	123 (99.1)	124 (100)	67 (54)	22 (17.7)	9 (7.2)	33 (26.6)	119 (95.9)	97 (78.3)	0 (0)	87 (70.1)	2 (1.6)	3 (2.4)

Note: Significant *p*-values (<0.05) are shown in bold.

**Table 3:** Distribution of virulence genes related to the resistance phenotype to β-lactamase inhibitor antibiotics and the β-lactamase genotype in strains isolated from healthcare-associated infections.

		Healthcare-Associated Infections (n = 67)							
		Protease	Phospholipases		Elastases	Rhamnolipids		Quorum-Sensing	
		*aprA* No. (%)	*pIcH* No. (%)	*plcN* No. (%)	*lasA* No. (%)	*lasB* No. (%)	*rhLA* No. (%)	*lasI* No. (%)	*rhII* No. (%)
Phenotype of β-lactamase inhibitor antibiotics	Amoxicillin/ clavulanic acid	64 (95.5)	66 (98.5)	61 (91)	65 (97)	46 (68.6)	66 (98.5)	66 (98.5)	66 (98.5)
	Ampicillin/ sulbactam	65 (97)	67 (100)	61 (91)	66 (98.5)	47 (70.1)	67 (100)	67 (100)	67 (100)
	Piperacillin/ tazobactam	34 (50.7)	34 (50.7)	31 (46.2)	33 (49.2)	26 (38.8)	34 (50.7)	34 (50.7)	34 (50.7)
β-lactamase genes	*bla_SHV_*	12 (17.9)	12 (17.9)	11 (16.4)	12 (17.9)	8 (11.9)	12 (17.9)	12 (17.9)	12 (17.9)
	*bla_CITM_*	1 (1.4)	1 (1.4)	1 (1.4)	1 (1.4)	1 (1.4)	1 (1.4)	1 (1.4)	1 (1.4)
	*bla_OXA-_*_1_	14 (20.8)	15 (22.3)	13 (19.4)	15 (22.3)	10 (14.9)	15 (22.3)	15 (22.3)	15 (22.3)
	*bla_OXA-_*_2_	63 (94)	65 (97)	59 (88)	64 (95.5)	47 (70.1)	65 (97)	65 (97)	65 (97)
	*bla_OXA-_*_4_	52 (77.6)	54 (80.5)	49 (73.1)	53 (79.1)	37 (55.2)	54 (80.5)	54 (80.5)	54 (80.5)
	*bla_OXA-_*_10_	0 (0)	0 (0)	0 (0)	0 (0)	0 (0)	0 (0)	0 (0)	0 (0)
	*bla_GES_*	49 (73.1)	50 (74.6)	45 (67.1)	50 (74.6)	41 (61.1)	50 (74.6)	50 (74.6)	50 (74.6)
	*bla_PER_*	0 (0)	1 (1.4)	0 (0)	1 (1.4)	1 (1.4)	1 (1.4)	1 (1.4)	1 (1.4)
	*bla_VEB_*	0 (0)	2 (2.9)	2 (2.9)	2 (2.9)	2 (2.9)	2 (2.9)	2 (2.9)	2 (2.9)
	*p*-value	**0.0004**	**0.0004**	**0.0004**	**0.0004**	**0.0004**	**0.0004**	**0.0004**	**0.0004**

Note: Significant *p*-values (<0.05) are shown in bold.

**Table 4:** Distribution of virulence genes related to the resistance phenotype to β-lactamase inhibitor antibiotics and the β-lactamase genotype in strains isolated from community-acquired infections.

		Community-Acquired Infections (n = 57)							
		Protease	Phospholipases		Elastases		Rhamnolipids	Quorum-Sensing	
		*aprA* No. (%)	*pIcH* No. (%)	*plcN* No. (%)	*lasA* No. (%)	*lasB* No. (%)	*rhLA* No. (%)	*lasI* No. (%)	*rhII* No. (%)
Phenotype of β-lactamase inhibitor antibiotics	Amoxicillin/ clavulanic acid	56 (98.2)	57 (100)	51 (89.4)	56 (98.2)	49 (85.9)	57 (100)	57 (100)	57 (100)
	Ampicillin/ sulbactam	56 (98.2)	57 (100)	51 (89.4)	56 (98.2)	49 (85.9)	57 (100)	57 (100)	57 (100)
	Piperacillin/ tazobactam	33 (57.8)	24 (42.1)	20 (35)	33 (57.8)	31 (54.3)	24 (42.1)	33 (57.8)	33 (57.8)
β-lactamase genes	*bla_SHV_*	10 (17.5)	10 (17.5)	10 (17.5)	10 (17.5)	8 (14)	10 (17.5)	10 (17.5)	10 (17.5)
	*bla_CITM_*	8 (14)	8 (14)	7 (12.2)	8 (14)	7 (12.2)	8 (14)	8 (14)	8 (14)
	*bla_OXA-_*_1_	18 (31.5)	18 (31.5)	15 (26.3)	18 (31.5)	16 (28.0)	18 (31.5)	18 (31.5)	18 (31.5)
	*bla_OXA-_*_2_	54 (94.7)	55 (96.4)	49 (85.9)	54 (94.7)	46 (80.7)	55 (96.4)	55 (96.4)	55 (96.4)
	*bla_OXA-_*_4_	45 (78.9)	45 (78.9)	40 (70.1)	44 (77.1)	37 (64.9)	45 (78.9)	45 (78.9)	45 (78.9)
	*bla_OXA-_*_10_	0 (0)	0 (0)	0 (0)	0 (0)	0 (0)	0 (0)	0 (0)	0 (0)
	*bla_GES_*	36 (63.1)	37 (64.9)	31 (54.3)	37 (64.9)	30 (52.6)	37 (64.9)	37 (64.9)	37 (64.9)
	*bla_PER_*	1 (1.7)	1 (1.7)	1 (1.7)	1 (1.7)	1 (1.7)	1 (1.7)	1 (1.7)	1 (1.7)
	*bla_VEB_*	1 (1.7)	1 (1.7)	1 (1.7)	1 (1.7)	1 (1.7)	1 (1.7)	1 (1.7)	1 (1.7)
	*p*-value	**0.0004**	**0.0004**	**0.0004**	**0.0004**	**0.0004**	**0.0004**	**0.0004**	**0.0004**

Note: Significant *p*-values (<0.05) are shown in bold.

**Table 5:** Distribution of phylogroups and MIC to β-lactamase inhibitor antibiotics in strains.

Strain Origin (n = 124)	Infection	Phylogroup			MIC for β-Lactamase Inhibitor Antibiotics (0.016–256 μg/mL)			
		1 No. (%)	2 No. (%)	Not Determined No. (%)	Ticarcillin/ Clavulanic Acid		Piperacillin/ Tazobactam	
					MIC	No. (%)	MIC	No. (%)
Healthcare associated infections (n = 67)	Pneumonia (n = 16)	3 (18.7)	1 (6.2)	12 (75)	>256	16 (100)	>256 48 32 24 6 4 3	6 (37.5) 1 (6.2) 2 (12.5) 2 (12.5) 3 (18.7) 1 (6.2) 1 (6.2)
	Bacteremia (n = 24)	4 (16.6)	2 (8.3)	18 (75)	>256 64	23 (95.8) 1 (4.1)	>256 48 32 24 16 12 8	9 (37.5) 4 (16.6) 4 (16.6) 2 (8.3) 2 (8.3) 2 (8.3) 1 (4.1)
	Wound infection (n = 27)	4 (14.8)	2 (7.4)	21 (77.7)	>256	27 (100)	>256 64 48 32 24 12 3	18 (66.6) 1 (3.7) 1 (3.7) 1 (3.7) 1 (3.7) 2 (7.4) 3 (11.1)
Community-acquired infections (n = 57)	Respiratory infection (n = 10)	1 (10)	1 (10)	8 (80)	>256	10 (100)	>256 16 12 8	7 (70) 1 (10) 1 (10) 1 (10)
	Catheter associated infection (n = 10)	2 (20)	2 (20)	6 (60)	>256	10 (100)	>256 96 48	8 (80) 1 (10) 1 (10)
	UTI (n = 37)	8 (21.6)	0 (0)	29 (78.3)	>256	37 (100)	>256 64 48 32 28 16 12 8 4	19 (51.3) 1 (2.7) 2 (5.4) 4 (10.8) 1 (2.7) 2 (5.4) 1 (2.7) 6 (16.2) 1 (2.7)
	Total, No. (%)	22 (17.7)	8 (6.4)	94 (75.8)	>256 (R) 64 (S)	123 (99.1) 1 (0.9)	>256 (R) 3–96 (S)	67 (54) 57 (46)

Note: R = Resistant, S = Susceptible.

**Table 6:** Patterns of association of virulence and β-lactamase genes with the phenotype of resistance to β-lactamase inhibitor antibiotics, and with phylogroups in strains.

Pattern Number	Genotype Combination Related to Inhibitor Resistance Phenotype	Healthcare Associated Infections (n = 67)			Community-Acquired Infections (n = 57)			Phylogroup			Total (n = 124) No. (%)
		Pneumonia (n = 16) No. (%)	Bacteremia (n = 24) No. (%)	Wound Infection (n = 27) No. (%)	Respiratory Infection (n = 10) No. (%)	Catheter Associated Infection (n = 10) No. (%)	UTI (n = 37) No. (%)	1	2	No Deter Mined	
1	*aprA*/*pIcH*/*pIcN*/*lasA*/*lasB*/*rhLA*/*lasI*/*rhII*/*bla_OXA-_*_2_/*bla_OXA-_*_4_/*bla_GES_*/*Am-Sul*/*Amox-Ac.Clavul*/*Piper-Tazo*	2 (12.5)	3 (12.5)	5 (18.5)	2 (20)	0 (0)	2 (5.4)	2	1	11	14 (11.3)
2	*aprA*/*pIcH*/*pIcN*/*lasA*/*lasB*/*rhLA*/*lasI*/*rhII*/*bla_OXA-_*_2_/*bla_OXA-_*_4_/*Am-Sul*/*Amox-Ac.Clavul*/*Piper-Tazo*	0 (0)	1 (4.1)	0 (0)	2 (20)	2 (20)	5 (13.5)	2	1	7	10 (8.0)
3	*aprA*/*pIcH*/*pIcN*/*lasA*/*lasB*/*rhLA*/*lasI*/*rhII*/*bla_OXA-_*_2_/*bla_OXA-_*_4_/*bla_GES_*/*bla_OXA-_*_1_/*Am-Sul*/*Amox-Ac.Clavul*/*Piper-Tazo*	0 (0)	0 (0)	3 (11.1)	1 (10)	1 (10)	4 (10.8)	3	0	6	9 (7.2)
4	*aprA*/*pIcH*/*pIcN*/*lasA*/*lasB*/*rhLA*/*lasI*/*rhII*/*bla_OXA-2_*/*bla_OXA-_*_4_/*bla_GES_*/*Am-Sul*/*Amox-Ac.Clavul*	2 (12.5)	2 (8.3)	3 (11.1)	0 (0)	0 (0)	1 (2.7)	1	0	7	8 (6.4)
5	*aprA*/*pIcH*/*pIcN*/*lasA*/*lasB*/*rhLA*/*lasI*/*rhII*/*bla_OXA-_*_2_/*bla_GES_*/*Am-Sul*/*Amox-Ac.Clavul*/*Piper-Tazo*	0 (0)	1 (4.1)	1 (3.7)	0 (0)	0 (0)	3 (8.1)	0	0	5	5 (4.0)
6	*aprA*/*pIcH*/*pIcN*/*lasA*/*rhLA*/*lasI*/*rhII*/*bla_OXA-_*_2_/*bla_OXA-_*_4_/*bla_GES_*/*Am-Sul*/*Amox-Ac.Clavul*/*Piper-Tazo*	0 (0)	2 (8.3)	2 (7.4)	0 (0)	1 (10)	0 (0)	1	0	4	5 (4.0)
7	*aprA*/*pIcH*/*pIcN*/*lasA*/*rhLA*/*lasI*/*rhII*/*bla_OXA-_*_2_/*bla_OXA-_*_4_/*bla_GES_*/*Am-Sul*/*Amox-Ac.Clavul*	0 (0)	1 (4.1)	2 (7.4)	0 (0)	0 (0)	2 (5.4)	0	0	5	5 (4.0)
8	*aprA*/*pIcH*/*pIcN*/*lasA*/*lasB*/*rhLA*/*lasI*/*rhII*/*bla_OXA-_*_2_/*bla_GES_*/*Am-Sul*/*Amox-Ac.Clavul*	2 (12.5)	1 (4.1)	0 (0)	1 (10)	0 (0)	0 (0)	2	1	1	4 (3.2)
9	*aprA*/*pIcH*/*lasA*/*lasB*/*rhLA*/*lasI*/*rhII*/*bla_OXA-_*_2_/*bla_OXA-_*_4_/*bla_GES_*/*Am-Sul*/*Amox-Ac.Clavul*	1 (6.2)	1 (4.1)	0 (0)	0 (0)	0 (0)	1 (2.7)	0	0	3	3 (2.4)
10	*aprA*/*pIcH*/*pIcN*/*lasA*/*lasB*/*rhLA*/*lasI*/*rhII*/*bla_OXA-_*_2_/*Am-Sul*/*Amox-Ac.Clavul*/*Piper-Tazo*	2 (12.5)	0 (0)	0 (0)	0 (0)	1 (10)	0 (0)	1	0	2	3 (2.4)
11	*aprA*/*pIcH*/*lasA*/*lasB*/*rhLA*/*lasI*/*rhII*/*bla_OXA-_*_2_/*bla_OXA-_*_4_/*bla_GES_*/*bla_OXA-_*_1_/*Am-Sul*/*Amox-Ac.Clavul*/*Piper-Tazo*	1 (6.2)	0 (0)	0 (0)	1 (10)	0 (0)	1 (2.7)	0	0	3	3 (2.4)
12	*aprA*/*pIcH*/*pIcN*/*lasA*/*rhLA*/*lasI*/*rhII*/*bla_OXA-_*_2_/*bla_OXA-_*_4_/*Am-Sul*/*Amox-Ac.Clavul*/*Piper-Tazo*	1 (6.2)	1 (4.1)	1 (3.7)	0 (0)	0 (0)	0 (0)	1	0	2	3 (2.4)
13	*aprA*/*pIcH*/*pIcN*/*lasA*/*lasB*/*rhLA*/*lasI*/*rhII*/*bla_OXA-_*_2_/*bla_OXA-_*_4_/*bla_GES_*/*bla_OXA-_*_1_/*Am-Sul*/*Amox-Ac.Clavul*	0 (0)	0 (0)	2 (7.4)	0 (0)	0 (0)	1 (2.7)	2	0	1	3 (2.4)
14	*aprA*/*pIcH*/*pIcN*/*lasA*/*lasB*/*rhLA*/*lasI*/*rhII*/*bla_OXA-_*_2_/*bla_OXA-_*_4_/*bla_GES_*/*bla_OXA-_*_1_/*bla_SHV_*/*Am-Sul*/*Amox-Ac.Clavul*/*PiperTazo*	0 (0)	0 (0)	1 (3.7)	0 (0)	0 (0)	1 (2.7)	0	0	2	2 (1.6)
15	*aprA*/*pIcH*/*pIcN*/*lasA*/*l*/*rhLA*/*lasI*/*rhII*/*bla_OXA-_*_2_/*Am-Sul*/*Amox-Ac.Clavul*	0 (0)	1 (4.1)	0 (0)	0 (0)	0 (0)	1 (2.7)	0	0	2	2 (1.6)
16	*aprA*/*pIcH*/*pIcN*/*lasA*/*lasB*/*rhLA*/*lasI*/*rhII*/*bla_OXA-_*_2_/*Am-Sul*/*Amox-Ac.Clavul*	0 (0)	1 (4.1)	0 (0)	1 (10)	0 (0)	0 (0)	0	0	2	2 (1.6)
17	*aprA*/*pIcH*/*pIcN*/*lasA*/*lasB*/*rhLA*/*lasI*/*rhII*/*bla_OXA-_*_2_/*bla_OXA-_*_4_/*bla_GES_*/*bla_OXA-_*_1_/*bla_SHV_*/*Am-Sul*/*Amox-Ac.Clavul*	1 (6.2)	0 (0)	0 (0)	0 (0)	0 (0)	1 (2.7)	0	1	1	2 (1.6)
18	*aprA*/*pIcH*/*pIcN*/*lasA*/*rhLA*/*lasI*/*rhII*/*bla_OXA-_*_2_/*bla_OXA-_*_4_/*bla_GES_*/*bla_OXA-_*_1_/*bla_SHV_*/*Am-Sul*/*Amox-Ac.Clavul*	0 (0)	1 (4.1)	0 (0)	0 (0)	0 (0)	1 (2.7)	0	0	2	2 (1.6)
19	*aprA*/*pIcH*/*pIcN*/*lasA*/*rhLA*/*lasI*/*rhII*/*bla_OXA-_*_2_/*bla_OXA-_*_4_/*bla_GES_*/*bla_OXA-_*_1_/*Am-Sul*/*Amox-Ac.Clavul*	0 (0)	1 (4.1)	0 (0)	0 (0)	0 (0)	1 (2.7)	0	1	1	2 (1.6)
19–55	Other combinations	4 (25)	7 (29.1)	7 (25.9)	2 (20)	5 (50)	12 (32.4)	7	3	27	37 (29.8)

## Data Availability

The original contributions presented in the study are included in the article.
